# Branched chain amino acids prime metabolic inflammation

**DOI:** 10.1016/j.molmet.2025.102308

**Published:** 2025-12-15

**Authors:** Nandini K. Doshi, Tristan Pesaresi, Trishya Pagadala, William Dion, Yang Zhang, Natalie L. David, Tânia Amorim, Wenjia Wang, G.V. Naveen Kumar, Bokai Zhu, Silvia Liu, Parth Patwari, Pouneh K. Fazeli, Matthew L. Steinhauser

**Affiliations:** 1 Aging Institute of UPMC and University of Pittsburgh School of Medicine, Pittsburgh PA, USA; 2 Division of Genetics, Department of Medicine, Brigham and Women's Hospital and Harvard Medical School, Boston MA, USA; 3 School of Public Health (Shenzhen), Sun Yat-sen University, Shenzhen, Guangdong, China; 4 Center for Human Integrative Physiology, University of Pittsburgh School of Medicine, Pittsburgh PA, USA; 5 Neuroendocrinology Unit, Division of Endocrinology and Metabolism, Department of Medicine, University of Pittsburgh School of Medicine, Pittsburgh PA, USA; 6 Department of Biostatistics, School of Public Health, University of Pittsburgh, USA; 7 Pittsburgh Liver Research Center, University of Pittsburgh School of Medicine, USA; 8 Organ Pathobiology and Therapeutics Institute, University of Pittsburgh School of Medicine, USA; 9 Department of Pharmacology and Chemical Biology, University of Pittsburgh School of Medicine, USA; 10 Division of Cardiovascular Medicine, Brigham and Women's Hospital and Harvard Medical School, Boston, MA, USA; 11 Takeda Pharmaceuticals, Cambridge MA, USA; 12 Division of Cardiology, Department of Medicine, University of Pittsburgh School of Medicine, Pittsburgh, Pennsylvania, USA

**Keywords:** Fasting, Amino acids, Inflammation, Endotoxin, Obesity

## Abstract

Sterile inflammation is associated with a broad range of metabolic stressors including both dietary excess and prolonged fasting. In a 10-day human fasting study, we previously identified a surge in the circulating inflammatory biomarker, C-reactive protein (CRP), which we leveraged in the current study to identify novel metabolic inflammatory correlates. With a variety of longitudinal metabolic variables as input, including metabolomics, we identified branched chain amino acids (BCAA) as the top candidate inflammatory correlate. We then used *in vitro* myeloid/macrophage culture and *in vivo* murine models to test BCAA as a determinant of inflammatory signaling. Short-term exposure to BCAA alone had modest effects on a variety of immune readouts; however, when coupled with a second stimulus, such as exposure to endotoxin or when administered to diet-induced obese mice, members of the JAK/STAT/cytokine signaling pathways were augmented on the transcriptional level by concurrent BCAA administration in multiple tissues, including visceral adipose and liver. The modifying effect of BCAA on inflammatory stressors translated into increased levels of circulating inflammatory cytokines. Collectively, these data position BCAA as an immune priming factor, a potential mechanism underlying the well-established association between circulating BCAA and diverse diseases of aging.

## Highlights

• CRP surge with human fasting used to screen for candidate immuno-metabolic drivers.
• Branched chain amino acids identified as top CRP correlate with human fasting.
• Branched chain amino acids reprogram chromatin landscape in murine liver.
• Branched chain amino acids potentiate endotoxin responses in mice.
• Branched chain amino acids augment inflammatory programs in obese mice.

## 1 Introduction

The emerging field of immunometabolism seeks to define and contextualize complex bidirectional relationships between immune function and metabolic processes. Immune cells depend on distinct metabolic pathways to support their activation, differentiation, and cytokine production [1,2]. Conversely, systemic and local metabolic environments dynamically influence immune cell activity, especially during states of stress and infection [[3], [4], [5]]. Inflammation, traditionally considered solely a response to injury or infection, also arises in response to metabolic stress or tissue dysfunction, even in the absence of infection [[6], [7], [8]]. Myeloid cells, including macrophages and dendritic cells, are especially responsive to these cues during the innate inflammatory response resulting in specialized stress responses [9,10]. Consequently, sterile inflammation is a common pathophysiological feature of a diverse array of chronic diseases, including atherosclerosis, type 2 diabetes mellitus (T2D), cancer, Alzheimer's disease, amongst others [[11], [12], [13]].

Nutritional stress is a critical factor in sterile inflammatory responses, best exemplified by states of caloric excess and obesity where dysregulated immune signaling contributes to systemic insulin resistance [14]. Chronic inflammation in these settings is not a byproduct of infection, but is driven by persistent metabolic imbalance, where nutrient excess acts as a sterile inflammatory trigger [15]. This sustained immunometabolic disturbance impairs the resolution of inflammation, creating a feedback loop that reinforces both immune activation and metabolic dysfunction [16]. The complete absence of caloric intake with fasting—the energetic converse to caloric excess—also represents a state of metabolic stress. While there may be beneficial effects of intermittent exposure to the metabolic stress of fasting with the so-called ‘hormesis’ effect of fasting having gained attention for possible health benefits, the fasting response is not simply the counter-opposite to caloric excess [17]. Indeed, the induction of sterile inflammation is not exclusive to obesity, as we recently discovered in longitudinal multi-omics analyses of humans undergoing a 10-day zero-calorie fast [18,19]. Fasting induces an inflammatory transcriptional program in adipose tissue and a systemic surge in circulating inflammatory biomarkers, including C-reactive protein (CRP) [19].

In the present study, we leveraged our previously published longitudinal fasting dataset inclusive of metabolomics and clinical measures to uncover novel correlates to the inflammatory biomarker CRP [[18], [19], [20]]. Our *a priori* hypothesis was that any such list of putative regulators of metabolic inflammation would be dominated by lipid metabolites, given the importance of lipid metabolism to the adaptive fasting response and precedent for various lipids and lipid catabolites as bioactive modulators of inflammation. While lipid species were indeed represented, we unexpectedly identified branched chain amino acids (BCAA) as the top CRP correlate and candidate modulator of metabolic inflammation. We utilized myeloid derived inflammatory cell lines*, in vitro,* and murine studies to directly test the inflammatory properties of BCAA in the context of both acute and chronic inflammatory models, integrating mRNA, epigenomic (ChIP-seq), and cytokine responses as readouts for immuno-metabolic reprogramming. While exposure to increased concentrations of BCAA over a period of hours to several days modestly impacted a subset of immune/inflammatory markers, a modifying effect of BCAA was most evident with exposure to a secondary inflammatory stimulus. Therefore, the merging of an unbiased human screen with reductionist validation in cell and murine models identified a previously unappreciated BCAA-inflammatory link, suggesting that tonically elevated levels of BCAA may predispose to inflammation through an immune-priming mechanism.

## 2 Results

### 2.1 Identification of BCAAs as candidate modulators of metabolic inflammation

**Figure 1:**
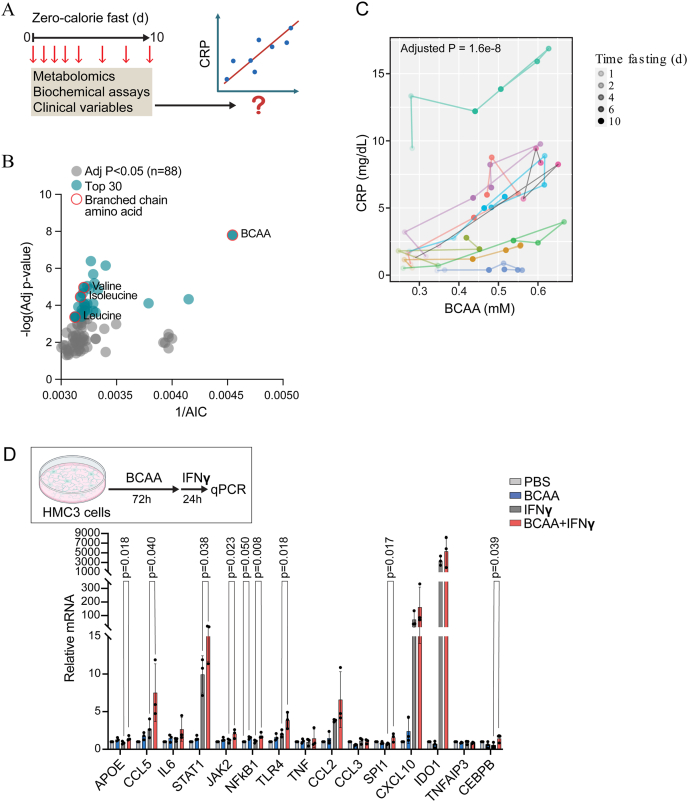
**Identification of branched chain amino acids as a candidate regulator of metabolic inflammation**. A. Schematic of study design. Metabolomics variables and other clinical phenotyping variables collected during 10-day zero calorie inpatient fast. Longitudinal analyses of the time series data were performed to identify correlates to serum C-reactive protein (CRP). B. Significant CRP correlates (n = 88 adjusted p < 0.05). The top 30 ranked by -log (Adj p-value) are indicated in blue (y-axis). The x-axis indicates 1 over the Akaike information criterion (AIC), such that a higher value is indicative of better fit. C. Top CRP correlate, branched chain amino acids (BCAA), measured by biochemical assay. Individual BCAA measurements (X-axis) are plotted in relation to CRP measurements (Y-axis). Each participant is plotted using a different color, with the dots ranging from unfilled to solid as a function of the duration of fasting. D. Inflammatory gene expression of HMC3 cells cultured in BCAA-enriched media (5 mM) for 72 h followed by IFNγ (10 ng/mL) for 24 h (n = 3 biological replicates). Data normalized to GAPDH and control (PBS) values and analyzed with one-way ANOVA with Šidák multiple comparisons test.

We previously demonstrated that prolonged fasting in humans drives a sterile inflammatory response, which we leveraged in the current study to identify potential novel regulators of metabolic inflammation [19]. To gain more insight into potential regulators of this sterile inflammatory response, we conducted an unbiased exploratory analysis to identify longitudinal correlates to C-reactive protein (CRP), a biomarker of inflammation, previously shown to surge in circulation over 10 days of a zero-calorie fast (Figure 1A). Potential correlates included the longitudinal set of previously published metabolomics analyses in addition to a battery of factors measured by targeted biochemical or protein/hormone assay. Interestingly, we found that branched chain amino acids (BCAA), measured with a biochemical assay, was the top CRP correlate (Figure 1B,C). The BCAAs (valine, isoleucine, and leucine) were also measured individually as a part of the metabolomics analysis, each of which were also independently amongst the top 30 CRP correlates (Table 1). Based on this result, we hypothesized that BCAAs modulate inflammation.

**Table 1:** Longitudinal correlates to C-reactive protein during 10-day fast.

Rank	Correlate variable	AIC	Adj p-value
1	BCAA	220	0.000000016
2	C36.4.PC.B	306	0.000000405
3	C18.2.SM	294	0.000000709
4	C56.8.TAG	304	0.000002077
5	C56.9.TAG	303	0.000006447
6	C40.6.PC	309	0.000009064
7	Valine	312	0.000010951
8	C18.1.SM	307	0.000013092
9	C36.2.PS.plasmalogen	300	0.000014431
10	C38.6.PC	313	0.000022446
11	Isoleucine	315	0.000034816
12	Total protein	241	0.000046786
13	C34.3.PE.plasmalogen	264	0.000077623
14	C36.4.PE.plasmalogen	304	0.000080284
15	C24.0.SM	312	0.000080941
16	C58.9.TAG	309	0.000082432
17	C40.9.PC	311	0.00009079
18	C38.4.PC	317	0.000118797
19	C58.10.TAG	311	0.000122499
20	C18.0.SM	314	0.000177555
21	C56.7.TAG	311	0.000185927
22	C58.11.TAG	309	0.000188097
23	C60.12.TAG	311	0.00019047
24	C18.2.LPE	304	0.000196941
25	C18.1.LPE	302	0.000240943
26	C22.0.SM	314	0.000260773
27	C14.0.SM	313	0.000306003
28	Alloisoleucine	320	0.000392386
29	C18.2.LPC	314	0.000409944
30	Leucine	320	0.000430265

Tissue macrophages are a critical cell type involved in regulating innate inflammatory responses to pathogens and metabolic stressors. Therefore, as a first test of a potential BCAA-mediated effect on inflammatory pathways, we assessed the modulatory effect of BCAA on canonical inflammatory stimuli in a series of macrophage-like cell lines: RAW264.7, THP-1, and HMC3 cells. We cultured cells for 72 h in BCAA-enriched or control media prior to acute inflammatory stressors. Neither RAW nor THP-1 cells exhibited a consistent BCAA effect on the transcriptional response to endotoxin across biological replicates even though select experiments suggested a modulatory effect (Figure S1). By contrast, HMC3 culture in BCAA potentiated the transcription of several inflammatory genes when cells were exposed to either endotoxin or the metabolic stressor palmitic acid, including *JAK2*, and *CCL5* (Figure S2). HMC3 cells also exhibit stereotypical responses to damage-associated molecular programs, including those propagated by interferon (IFN)γ, providing rationale to further test for a BCAA modulatory effect on the IFNγ response. In this context, BCAA augmented the transcriptional response of *APOE*, *CCL5, STAT1, JAK2, NFKB1, TLR4,* and *CEBPB,* with a trend towards augmented *IL6* and *CCL2* (Figure 1D). An additional testing of a subset of amino acids with diverse metabolic properties did not replicate this effect, arguing against a non-specific amino acid effect (Figure S2). Given that elevated levels of amino acids may activate nutrient sensitive mTOR signaling, we further tested potential modulatory effects of the mTOR inhibitor rapamycin, focusing on the most robustly regulated genes (*STAT1, TLR4, CCL5*). In this context, rapamycin treatment concurrent with BCAA elevation did not attenuate the BCAA effects on the expression of these specific genes (Figure S2). Across all three cell lines, there was minimal effect of BCAA exposure alone arguing against a direct pro-inflammatory effect of BCAA; however, these data in a macrophage-like cell line suggest that tonic exposure to elevated BCAA may potentiate responses to classical inflammatory stimuli and provided rationale to prioritize BCAA for further *in vivo* study.

### 2.2 BCAA modulate the acute inflammatory response to endotoxin in mice

We next aimed to directly test whether BCAA may stimulate inflammatory signaling, *in vivo.* We sought an approach to elevating systemic BCAA in an acute (hours) to subacute (days) timeframe, testing whether a single intraperitoneal injection of BCAA was sufficient to elevate systemic levels, as measured by biochemical assay in plasma. A dose of 9 mg resulted in an ∼4X increase in BCAA levels at 15min that remained detectable above baseline levels (∼1.4X) at 8 h but was back to baseline by 24 h post-injection (Figure S3). As an initial test of an acute BCAA effect, *in vivo,* we sacrificed C57BL/6 mice 4 h after intraperitoneal injection with BCAA or PBS (control) and performed peritoneal washing to obtain a cell population enriched in peritoneal macrophages. We measured mRNA expression of both inflammatory genes and a subset of known markers of macrophage polarization. Similar to what we observed in culture cells, BCAA alone did not modulate expression of the measured canonical inflammatory genes, including *Ifng* and *Tnf*; however, short-term BCAA exposure modestly enhanced mRNA expression of *Cd68* and *Cd163*, genes related to macrophage cell state (Figure S3).

**Figure 2:**
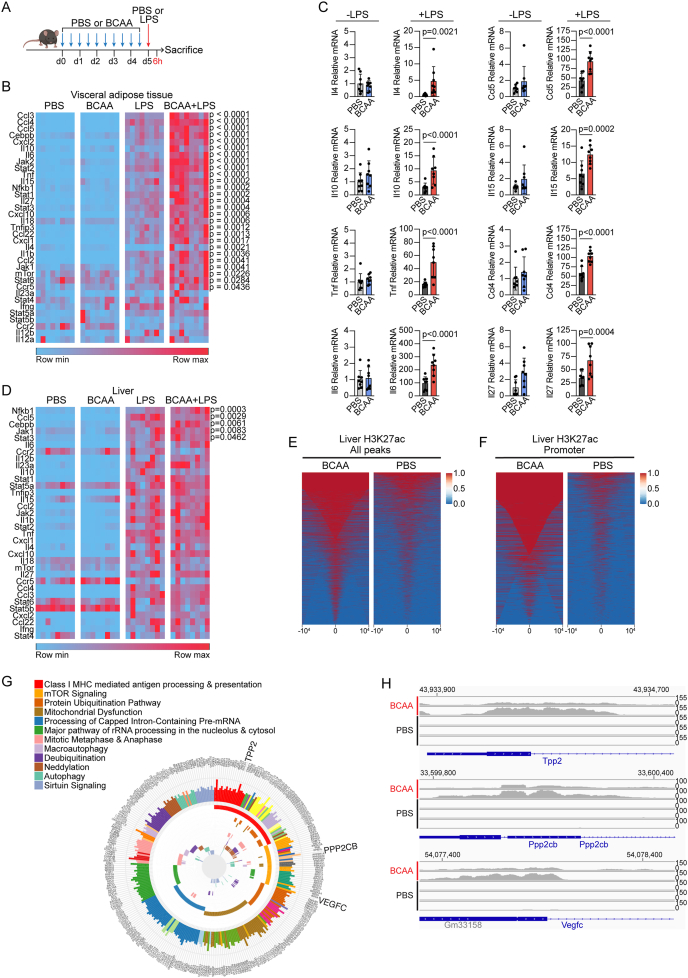
**BCAA modulate acute inflammatory responses to endotoxin**. A. Schematic depicting experimental design. Male C57Bl6 mice (n = 8) were administered BCAA or PBS (control) for 5 days by intraperitoneal injection followed by a single preterminal injection of PBA or LPS (0.1 mg/kg). B. Heatmap showing qPCR analysis of a targeted set of inflammation genes in visceral adipose tissue. Each square represents a biological replicate. Data normalized to *GAPDH* and control (PBS) values and significance assessed with one-way ANOVA with Šidák adjustment for two *a priori* comparisons to assess modifying effect of BCAA (PBS v BCAA; LPS v BCAA + LPS). C. Subset of genes from B are shown as examples of amplification of inflammatory gene expression when BCAA administration precedes secondary LPS exposure. Each dot represents a biological replicate. D. Heatmap showing qPCR analysis of inflammation genes in liver, as in ‘B’. E. H3K27ac ChIP-seq from murine liver after either BCAA or PBS (control) with heat map showing total peak enrichment. F. H3K27ac ChIP-seq from murine liver after either BCAA or PBS (control) with heat map showing peak enrichment at gene promoters. G. Pathway enrichment (top 12) for BCAA sensitive H3K27ac peaks in liver. H. Gene tracks at 3 genes represented in the top two pathways in G.

Since our *in vitro* data pointed to a possible indirect effect of BCAA, we hypothesized that a more pronounced *in vivo* effect might be evident with a second stimulus, like prior studies that have found that metabolic environments can exacerbate host response to inflammatory stimuli [21]. We also reasoned that repeated BCAA administration, more closely corresponding to the time-scale of the fasting-CRP effect seen in our human study (several days), would be a more relevant test of the hypothesis. As such, we intraperitoneally injected mice with BCAA or PBS every 12 h for five days, guided by our pharmacokinetic test (Figure S3). On the fifth day, we administered preterminal LPS (0.1 mg/kg) or PBS (control) by i.p. injection 6 h before sacrifice and harvesting of tissue samples (Figure 2A). We focused our analyses on immunogenic tissues with resident macrophage populations such as visceral adipose tissue (perigonadal), spleen, and liver. As expected, LPS treatment caused upregulation at the mRNA level of nearly all the inflammatory genes examined. While there were minimal differences in inflammatory gene expression between PBS control and BCAA injected groups without LPS administration, expression of numerous inflammatory genes in visceral fat was amplified when BCAA administration preceded LPS, including genes coding members of JAK/STAT and cytokine signaling pathways (Figure 2B–C). Interestingly, a similar effect was observed for a subset of BCAA metabolic genes in VAT, including a more pronounced augmentation of *Protein phosphatase, magnesium-dependent 1K (Ppm1k)* and *Branched-chain keto acid dehydrogenase kinase (Bdk)* with combined exposure to BCAA and LPS in VAT (Figure S4). We also evaluated the expression of inflammatory genes in liver and spleen. As seen in visceral fat, minimal differences were detected between groups without LPS exposure; however, LPS exposure significantly augmented the expression of several inflammatory genes across both tissue types, albeit not to the same extent as adipose tissue (Figure 2D, S4).

Transcriptional reprogramming can be mediated through remodeling of the chromatin landscape. Given that liver was one tissue exhibiting evidence of a BCAA reprogramming effect and in addition is amenable to tissue chromatin immunoprecipitation (ChIP)-seq, we performed ChIP using antibodies targeting the H3K27-acetyl histone mark, known to enrich at regions of open chromatin corresponding to active gene promoters or cis-regulatory transcriptional enhancers (Figure S5). Our *a priori* hypothesis was that BCAA treatment would result in such remodeling of the cis-regulatory landscape around canonical inflammatory genes. While we observed global changes in H3K27 acetylation secondary to BCAA treatment (Figure 2EF, S5), there was no indication of epigenomic changes at the sites we originally suspected including the genes that were significantly modulated by BCAA in the liver (*Nfkb1, Jak1, Stat3, Ccl5, Cebpb*); however, we discovered evidence of BCAA-mediated chromatin remodeling at other loci, particularly in regions around promoters (Figure S5). We performed pathway analyses of the collective nearest genes to peaks exhibiting a BCAA effect, finding enrichment of immunometabolic pathways (Figure 2GH). Indeed, the topmost enriched pathway was ‘*Class I MHC mediated antigen processing and presentation’.* The canonical nutrient sensing *mTor Signaling* pathway, which also is implicated in immune regulation, was the second most enriched pathway. Collectively, these results suggest that BCAA treatment does not directly cause remodeling of H3K27 acetylation at promoters or enhancers of canonical inflammatory genes such as those encoding the cytokine effectors that were dynamically regulated in tissues and circulation, although it is important to note that we did not perform ChIP-seq of visceral adipose tissue where the BCAA modulatory effect was more pronounced. It is possible that there are modifications to the epigenome in addition to H3K27ac, including removal of repressive marks (*e.g.* H3Kme2), which may be sensitive to BCAA through the regulatory actions of alpha-ketoglutarate. Such possibilities could indicate an indirect priming mechanism mediated in part by chromatin remodeling at collaborating immunomodulators.

### 2.3 BCAA modulate tissue expression of macrophage markers

Given the predominance of macrophages as a critical immune cell type across the three examined tissues, coupled with their known plasticity and phenotypic heterogeneity as a function of their external microenvironment [22], we examined whether exposure to BCAA would modulate expression of markers associated with the classically activated M1 macrophage or alternatively activated M2 macrophage phenotypes. Across the three tissue types of interest, we observed marginal changes in the expression of M1 and M2 macrophage markers between the BCAA injected mice and control mice (Figure S6), suggesting that BCAA exposure alone did not induce a dramatic change in classical M1/2 metrics of macrophage phenotype.

As expected, endotoxin exposure (a known driver of M1 fate-specification) markedly augmented transcription of macrophage markers. We next tested whether BCAA further biased the endotoxin response towards a classical M1 phenotype. However, when we analyzed the modifying effect of BCAA in the context of endotoxin stimulus we observed significant amplification in gene expression of several cytokines, enzymes, and surface proteins that are classically associated with both the M1 (*Ido1, Tnf, Il6, Cd68, Cd64,* and *Il1b*) and the M2 (*Il4, Il10, Il18, Ccl22, Tgm2,* and *Tgfb1*) macrophage phenotype in visceral fat (Figure S6). Likewise, compared to LPS-injected mice, transcription of multiple genes associated with both M1 and M2 macrophage specification were augmented in spleen and liver harvested from mice injected with BCAA and LPS (Figure S6C-D). Taken together, these data suggest that while BCAA alone did not induce marked changes in tissue markers of macrophage phenotype, similar to prior analyses, BCAA administration was sufficient to augment expression of several markers across three tissues that contain resident macrophage populations without a specific M1/M2 preference.

### 2.4 BCAA augment systemic cytokine surge to endotoxin

**Figure 3:**
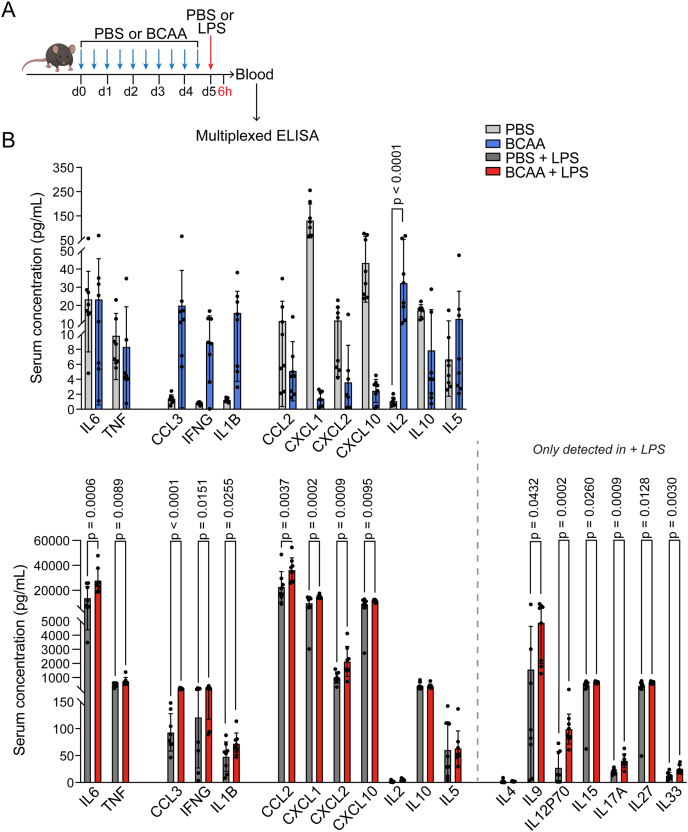
**BCAA augment systemic cytokine surge to endotoxin**. A. Schematic depicting experimental design. Male C57Bl6 mice (n = 8) were administered BCAA (9 mg/dose equimolar) or PBS (control) for 5 days followed by a single preterminal injection of PBS or LPS (0.1 mg/kg). Blood was analyzed by multi-plexed ELISA. B. Serum cytokine levels. Each dot represents a biological replicate. Significance assessed with one-way ANOVA with Šidák multiple comparisons test. Some cytokines were only detected in “+ LPS” groups. Significance between groups for those cytokines analyzed using unpaired t-tests.

Next, we sought to determine whether BCAA augmentation of inflammatory signaling at the transcriptional level in metabolically sensitive tissues would translate into alterations in circulating cytokines. To this end, we measured serum concentrations of cytokines by multi-plexed ELISA (Figure 3A). Notably, we observed an increase in cytokine levels, including CCL3, IFNG, IL1B, IL2, and IL5, even in the BCAA group without LPS exposure. The difference in circulating cytokine concentrations became even more pronounced between the BCAA and PBS groups with LPS exposure, with multiple cytokines being significantly upregulated in the BCAA + LPS group relative to PBS + LPS group (Figure 3B). Taken together, these findings suggest that while BCAA alone has a limited pro-inflammatory effect, BCAA potentiates both inflammatory gene expression and circulating serum cytokine concentration in response to a secondary inflammatory stimulus.

### 2.5 BCAA modulates inflammation in the diet-induced obesity model

**Figure 4:**
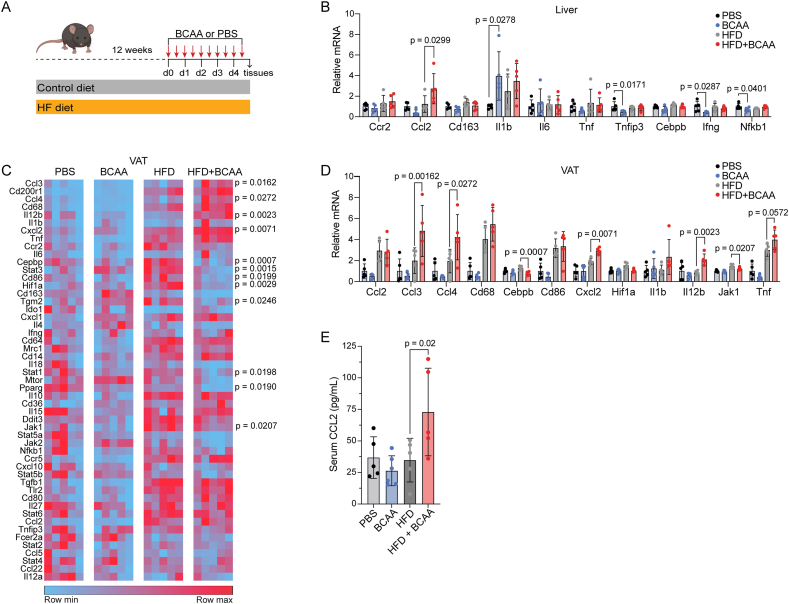
**Short-term BCAA administration modulates metabolic inflammatory responses in diet-induced obesity**. A. Schematic depicting experimental design (n = 5 mice). B. Inflammatory gene expression in liver. Each dot represents a biological replicate. Data was normalized to GAPDH and control (PBS) values and analyzed with one-way ANOVA with Šidák multiple comparisons test. C. Heat map showing qPCR analysis of a targeted set of inflammation genes in visceral adipose tissue. Each square represents a biological replicate. Data was normalized to GAPDH and control (PBS) values and analyzed with one-way ANOVA with Šidák multiple comparisons test. D. Subset of genes from C Inflammatory gene expression in visceral adipose tissue. Each dot represents a biological replicate. E. Serum CCL2 measured by ELISA (n = 5 mice PBS, HFD + PBS, and HFD + BCAA groups; n = 6 mice BCAA group). Significance assessed with one-way ANOVA with Šidák multiple comparisons test.

Chronic low-grade inflammation is a hallmark of obesity and type 2 diabetes (T2D) [6] and BCAA levels predict future T2D incidence [23]. The augmented response observed in BCAA injected mice after exposure to a secondary high grade inflammatory stimulus led us to question whether elevated BCAA could be immunomodulatory in obesity. We established the diet-induced obesity (DIO) model by feeding mice a high-fat diet (HFD) or standard chow control diet (SC) for twelve weeks prior. We again limited the BCAA exposure to 5 days by intraperitoneal injection with BCAA or PBS every 12 h for five days like the prior protocol, a timescale of relevance to our original fasting study but incongruent with the timescale of the DIO model (Figure 4A). As such, this experimental design tested a complementary immunomodulatory effect of short-term BCAA in the context of an established chronic inflammatory model, not the priming effect tested in prior experiments. We analyzed expression of cytokines and transcription factors associated with inflammation and markers of macrophage specification in both liver and visceral AT by qPCR. Compared to HFD mice, transcription of *Ccl2* was significantly augmented in liver of HFD mice also injected with BCAA (Figure 4B).

We evaluated the expression of the same panel of genes in visceral fat. Contrary to our prior experiment, we observed significant downregulation in the expression of multiple transcription factors associated with inflammation, such as *Jak1, Stat1, Stat3, Hif1a, Pparg,* and *Cebpb* (Figure 4C and D). Despite this, gene expression of multiple cytokines and chemokines, including *Ccl3, Ccl4, Il12b,* and *Cxcl2,* were significantly upregulated in visceral fat of HFD fed mice injected with BCAA relative to just HFD fed mice (Figure 4CD). We also measured serum concentration of CCL2. A minimal difference in CCL2 concentration was observed between the standard chow fed mice injected with BCAA relative to standard chow fed mice along; however, there was a significant increase in serum CCL2 concentration of HFD fed mice injected with BCAA relative to just HFD fed mice (Figure 4E). Collectively, these data suggest that the modulating effects of elevated systemic BCAA on inflammatory pathways extends to the chronic DIO disease model known to be characterized by low-grade sterile inflammation.

## 3 Discussion

Here we identified branched chain amino acids (BCAA) as a correlate to—and candidate enhancer of—sterile metabolic inflammation. We tested the immunomodulatory properties of BCAA in cellular and rodent inflammatory models, finding at most a modest effect of short term BCAA alone on inflammatory readouts; however, when paired with a secondary stimulus, BCAA potentiated cytokine signaling pathways. Although this study was launched based on a screen for candidate mediators of the inflammatory response to the metabolic stress of fasting in humans, experiments in murine endotoxemia and diet-induced obesity extend the potential relevance of this finding to broadly relevant pathophysiological states.

Our initial assumption when considering correlates to the fasting CRP surge was that such candidates would either be direct causal drivers of inflammatory pathways or simply correlate biomarkers, like CRP itself. The picture that emerged from these studies is more nuanced in suggesting that BCAA have an immunomodulatory effect that is exposed by a second stimulus. The observed magnitude of any such effect, however, was not as dramatic as some recently published examples where inflammatory outputs are synergistically amplified by dual agonism of canonical signaling pathways (*e.g.* IFN γ/TNFα or IL4/LPS) [24,25]. There is limited defined molecular infrastructure to activate potent inflammatory signal transduction pathways in response to metabolites such as BCAA, as occurs with receptor engagement of canonical inflammatory cytokines or recognition of pathogen or damage associated molecular patterns, nor is there an obvious adaptive explanation for why inflammatory pathways would be sensitive to an essential dietary amino acid. The closest such example of potential relevance is nutrient sensing mTOR signaling, the activation of which has complex effects on immunity and inflammation [26]. Indeed, our unbiased ChIP-seq analysis of liver tissue from mice administered a short course of supplemental BCAA implicated modulation of the chromatin landscape at mTOR pathway loci. In the subset of *in vitro* experiments where we assessed mTOR dependency with rapamycin, however, BCAA effects were not attenuated, suggesting the possibility of additional mTOR independent effects. Aside from mTOR signaling, BCAA are a potential fuel source for cells and energetic balance can impact inflammatory pathways [27,28]. In addition, byproducts of amino acid metabolism may have broad effects on the epigenomic regulatory architecture, either by regulating enzymatic modifiers of chromatin (*e.g.* alpha-ketoglutarate control of H3K9me2) or serving as substrate for such reactions (*e.g.* propionylation of histones using isoleucine derived propionyl-CoA) [29,30]. Therefore, while mTOR signaling may indeed be one factor underpinning BCAA modulatory effects, we posit that tonically elevated BCAA levels are likely to operate through multiple complex and context specific mechanisms, which may also explain why we did not see a consistent BCAA effect across all cell lines studied and why the BCAA effect was also generally more pronounced *in vivo.* Therefore, this study provides rationale for future more comprehensive analyses of the BCAA sensitive epigenomic landscape, including in adipose tissues where the most compelling BCAA modulatory effects were observed.

The potential relevance of the BCAA-inflammation link is underscored by a range of compelling epidemiological data. Cross-sectional analyses of the Women's Health study indicate associations between circulating BCAA and multiple inflammatory biomarkers, including CRP, fibrinogen, and soluble intercellular adhesion molecule-1 [31]. Similar analyses link BCAA levels to cardiometabolic disease, Alzheimer's disease, and cancer, in some instances BCAA predict future disease incidence raising the possibility of causality [23,[32], [33], [34], [35]]. One pathobiological process common across diseases linked to BCAA is the associated role of inflammation as a potential disease driver. Therefore, we speculate that tonically elevated BCAA participates in immune priming that sets the stage for disparate diseases of aging.

The starting point for this project was the sterile inflammatory response we observed with prolonged fasting in humans, a finding also seen in additional independent studies [36]. In identifying and validating BCAA as a lead CRP correlate and inflammatory modulator, the core observation that prolonged fasting is pro-inflammatory is strengthened. An open question is whether this sterile inflammatory response indicates fasting-mediated harm. Indeed, controlled exposure to the metabolic stress of fasting could trigger metabolic adaptations that are beneficial to health, so-called ‘hormesis.’ In addition, a key function of inflammatory responses, particularly those involving macrophages, is clearance of extracellular debris and pathological biomolecules, such as protein aggregates or toxic lipids, both of which are linked to aging or aging-related diseases [17]. Fasting is undertaken for variety of reasons, with or without a medical prescription, including religious/cultural practice, prior to surgical procedures, as therapy for certain medical conditions, as a weight loss measure, and to promote metabolic health. It will be critical for future studies to determine not only the context(s) where fasting is beneficial, but in addition how the duration and/or frequency of fasting exposures dictates whether the associated inflammatory stress response is adaptive or maladaptive.

Given that BCAA are essential amino acids, an important question relates to optimization of a metabolically healthy diet. The circulating levels of BCAA are not solely determined by dietary content, as evidenced by the well-described increase with prolonged fasting—i.e. no BCAA intake—that is largely attributed to flux from intracellular stores in skeletal muscle [18]. Genetic variants in genes involved in BCAA metabolism may also influence BCAA levels, with inborn errors of metabolism at the extreme [37]. Nonetheless, BCAA content in diet is also critical as indicated by both epidemiological analyses and careful prospective dietary studies in humans. Indeed, augmentation of leucine in the murine diet is sufficient to accelerate atherosclerotic plaque formation through mTOR signaling in macrophages [38,39]. While our study did not specifically investigate dietary BCAA—and therefore should be interpreted with caution—it does provide additional support for the emerging concept that dietary protein content and quality may be a determinant of pathobiological processes underpinning a range of chronic diseases of aging.

## 4 Methods

### 4.1 Human study

We studied healthy participants who underwent a 10-day, 0-calorie fasting protocol as previously published [20]. The human study was performed at the Massachusetts General Hospital and Brigham and Women's Hospital. The protocol was approved by the Mass Gen Brigham (formerly Partners HealthCare) Institutional Review Board and complied with the Health Insurance Portability and Accountability Act guidelines. Written informed consent was obtained from all participants. Briefly, research participants had normal thyroid function and women participants had regular menstrual cycles. Participants with a history of an eating disorder, diabetes mellitus, or other chronic illnesses were excluded. They were admitted to the Center for Clinical Investigation at Brigham and Women's Hospital in the morning after an overnight fast and remained inpatient for the duration of a 10 day fast and 1 day of supervised refeeding. The human data used for the longitudinal correlative analyses were previously published [[18], [19], [20]].

### 4.2 Murine studies

Animal experiments were approved by and in compliance with the Brigham and Women's Hospital Standing Committee on Animals or the University of Pittsburgh Institutional Animal Care and Use Committee. All mice used in this study were WT C57BL6/J and obtained from Jackson Laboratory. Mice were housed at 22 °C ± 2 °C, with a 12 h light (0700–1900 h), 12 h dark (1900–0700 h) cycle and *ad libitum* access to standard chow diet provided by animal facility and water. The diet-induced obesity (DIO) model was used as previously described with high fat (HFD) and standard chow control diets (CD) purchased from Research Diets (D12492 and D12450J) [40].

For the initial evaluation of the impact of BCAA on metabolic inflammation, mice received a single administration of BCAA or PBS via intraperitoneal (I.P.) injection followed by peritoneal macrophages (PeriMø) collection 4 h later. For the evaluation of multiple BCAA administrations on inflammatory response, mice received five days of BCAA or PBS treatment every 12 h via I.P. injection. To evaluate how BCAA modulates acute inflammatory response to endotoxin, 6 h after the last BCAA or PBS administration, a single preterminal dose of LPS (1 μg/kg) or PBS (control) injection was administered. Six hours later, tissues were harvested. The DIO model followed the same experimental protocol for BCAA or PBS injections, but mice had *ad libitum* access to either HFD or CD for the twelve weeks preceding and during the five-day period of BCAA administration.

Mice were sacrificed in carbon dioxide chamber with heart puncture as secondary form of euthanasia. Blood was collected in a microSST tube (BD), centrifuged at 1900 g × 15 min, serum collected and snap frozen. PeriMø were collected as described previously [41]. The peritoneal cavity was exposed, 10 mL of cold PBS injected, and then the PBS solution aspirated. Pelleted PeriMø were resuspended in 500 μL RNAzol (Molecular Research Company). All other tissues were snap frozen and stored in −80 °C until further use.

### 4.3 Biochemical and protein assays

Branched chain amino acid (BCAA) levels in serum were measured by branch chain amino acid detection kit (Abcam). Serum CCL3 (MIP-1a), CCL4 (MIP-2), CXCL1 (KC/GRO), CXCL10 (IP-10), IFNG, IL1B, IL2, IL4, IL5, IL6, IL9, IL10, IL12p70, IL15, IL17, IL27, IL33, and TNFA were measured using multiplex assay kits (Meso Scale Discovery K15245D and K15048D). Serum CCL2 (MCP-1) was measured using ELISA (R&D Systems MJE00B).

### 4.4 Reagents

BCAA solutions were prepared from pharmaceutical grade powders (Sigma–Aldrich, I7403, V0513, L8912) in PBS and sterile filtered. Endotoxin testing was performed to further confirm purity. Molarity of BCAA presented *in vitro* denotes a concentration to bring media to 5 mM sum of BCAA. Cell culture and IP lipopolysaccharide (LPS, Sigma–Aldrich, L4391) solutions were formulated in 1X PBS (Corning).

### 4.5 Cell culture

RAW 264.7 cells (ATCC TIB-71) were cultured in DMEM Glutamax supplemented with 10% Fetal Bovine Serum (FBS, Corning) and 1% penicillin/streptomycin (PS, Life Technologies) similar to previously described methods [42]. Cells were seeded in 6-well plates. Control media (CM) was added to half the wells and the other half were treated with BCAA-supplemented media. At the 48-hour time point, media was replenished. At 66-hour time point, lipopolysaccharide (LPS) or PBS as a control was added to the cells. Cells were collected in RNAzol or TRIzol at 6 h after addition.

THP-1 monocytes (ATCC TIB-202) were cultured in RPMI-60 (Corning) supplemented with 10% FBS (Corning) and 1% PS (Life Technologies) similar to previously described methods [19]. THP-1 cells were differentiated to macrophages as previously described, using phorbol 12-myristate 13-acetate (PMA, 100 ng/mL, Sigma–Aldrich) stimulation for 72 h, which was replenished at 48 h, at a starting concentration of 2 × 10^6^ cells/well in either 6-well or 12-well plates. THP-1 derived macrophages were switched to control media without PMA media for 24 h prior to initiation of experiments. At the initiation of experiments (hour 0), media was replenished such that control media (CM) was added to half the wells and the other half were treated with BCAA-supplemented media. At 48 h time point, media was replenished. At 66-hour time point, lipopolysaccharide (LPS) or PBS as a control was added to the cells. Cells were collected in RNAzol or TRIzol at 6 h after LPS addition. A total of four treatment groups were produced: CM, BCAA, CM + LPS, or BCAA + LPS.

Human microglial cells (HMC3, ATCC CRL-3304) were cultured in EMEM (ATCC, 30-30-2003) supplemented with 10% FBS (Gibco) and 1% PS (Gibco) following the ATCC protocol. Cells were seeded in 6-well plates at a starting concentration of 1 × 10^5^ cells/well and incubated for 24 h prior to initiation of experiments. At the initiation of the experiments (hour 0), media was replaced such that half the wells received control media and the other half of the wells received BCAA-supplemented media. At the 48 h time point, media was replenished in all wells with either control media or BCAA-supplemented media. At 72-hour time point, cells were treated with IFNγ (R&D) or PBS (vehicle).

### 4.6 Gene expression

Total RNA from cultured cells was isolated with either RNAzol (Molecular Research Company) or TRIzol (Invitrogen, 15-596-018) following the manufacturer's instructions. RNA from tissues was isolated using the Qiagen RNeasy kit, per the manufacturer's protocol. RNA yield and quality was confirmed by NanoDrop and cDNA synthesized (High-Capacity cDNA Reverse Transcription Kit; Thermo Fisher Scientific). qPCR was performed with Power Sybr Green PCR master mix (Applied Biosystems) using a QuantStudio 5 Real-Time PCR System and Quantstudio Design & Analysis Software (Applied Biosystems). Gene expression was normalized to GAPDH or Gapdh and fold change calculated by the ΔΔCt method. All undetermined reads were assigned a Ct value of 37.

### 4.7 Chromatin immunoprecipitation (ChIP)

ChIP for H3K27 acetylation was performed using the anti-histone H3 antibody (Acetyl-K27) from Abcam (ab4729) as previously described in reference [43] using the SimpleChIP Enzymatic Chromatin IP Kit (Cell Signaling Technology #9003) protocol. Briefly, mouse liver samples were cut into small pieces with scissors, fixed in PBS + 1.5% formaldehyde for 20 min, and the fixation reaction was quenched with glycine per the. Chromatin was isolated, enzymatically digested with MNase, and chromatin fragment size was measured on a 2% agarose gel. 5 μg of chromatin was used for IP, along with either 1 μg of the H3K27Ac antibody or 2 μg of normal Rabbit IgG (Cell Signaling Technology #2729, included in the aforementioned SimpleChIP Enzymatic Chromatin IP Kit). Complexes were washed, eluted from the beads with SDS buffer, and subjected to RNase and proteinase K treatment. Cross-linking was reversed by incubation overnight at 65 °C, and ChIP DNA was purified by phenol-chloroform extraction and ethanol precipitation.

Library generation and sequencing was performed by the University of Pittsburgh Health Sciences Sequencing Core (HSSC), Rangos Research Center, UPMC Children's Hospital of Pittsburgh, Pittsburgh, Pennsylvania, United States of America. ChIP enriched DNA was assessed for quality using an Agilent TapeStation 4150 and concentration was quantified on a Qubit FLEX fluorometer. Libraries were generated with the NEBNext Ultra II DNA prep kit (NEB: E7645S) according to the manufacturer's instructions. Briefly, 500 pg of input was used for each sample, with an adapter dilution of 1:25 for the adapter ligation, followed by 11 cycles of indexing PCR using NEBNext Multiplex Oligos for Illumina (NEB: E6440S). Library quantification and assessment was done using a Qubit FLEX fluorometer and an Agilent TapeStation 4150. Libraries were normalized and pooled to 2 nM by calculating the concentration based off the fragment size (base pairs) and the concentration (ng/μl) of the libraries. Sequencing was performed on an Illumina NextSeq 2000, using a P3 100 flow cell. The pooled library was loaded at 750 pM and sequencing was carried out with read lengths of 2 × 61 bp, with a target of 55–60 million reads per sample. Sequencing data was demultiplexed by the on-board Illumina DRAGEN FASTQ Generation software (v3.10.12).

ChIP-sequencing data analysis was performed as follows. The raw ChIP sequencing reads first underwent quality control and trimming by FastQC and Trimmomatic [43,44]. The high-quality reads were then aligned to the mouse reference genome mm10 using the Burrows-Wheeler Alignment tool (BWA) [45]. These reads were further filtered and marked duplicates by the SAMtools [46] and Picard tools (Broad Institute). Next, peaks were called using MACS [47], comparing the ChIP samples with their corresponding input libraries. Based on the above pre-processing steps, differential peaks and annotations were analyzed using R ‘DiffBind’ [48] and ‘ChIPseeker’ [49] packages. These binding regions were further visualized by the Integrative Genomics Viewer and used for the motif discovery and downstream pathway analysis. Data are available through the NCBI Gene Expression Omnibus (GSE289444).

### 4.8 Statistical analysis

Statistical analyses were performed with PRISM (Graphpad 9.0) or Rstudio (ver. 4.1.1). Student's t tests (two-sided) were used to compare two groups. For experiments containing three or more groups, we performed ANOVA. For skewed distributions, corresponding non-parametric tests were performed. Longitudinal analysis of the human fasting time series data to identify predictors of CRP was performed using a mixed-effects model in R. Potential predictors were included as fixed effects, and a random effect was fit for each participant. P values were adjusted for multiple comparisons with the Sidak method. A 2-sided *P* value of less than 0.05 was considered significant for all analyses.

## CRediT authorship contribution statement

**Nandini K. Doshi:** Writing – original draft, Visualization, Investigation, Formal analysis, Conceptualization. **Tristan Pesaresi:** Writing – review & editing, Investigation, Formal analysis, Conceptualization. **Trishya Pagadala:** Investigation. **William Dion:** Investigation, Formal analysis. **Yang Zhang:** Writing – review & editing, Investigation. **Natalie L. David:** Investigation. **Tânia Amorim:** Investigation. **Wenjia Wang:** Visualization, Formal analysis. **G.V. Naveen Kumar:** Investigation. **Bokai Zhu:** Writing – review & editing, Supervision. **Silvia Liu:** Supervision, Formal analysis. **Parth Patwari:** Writing – review & editing, Visualization, Formal analysis, Conceptualization. **Pouneh K. Fazeli:** Writing – review & editing, Supervision, Investigation, Conceptualization. **Matthew L. Steinhauser:** Writing – original draft, Visualization, Supervision, Investigation, Funding acquisition, Formal analysis, Conceptualization.

## Declaration of competing interest

The authors declare the following financial interests/personal relationships which may be considered as potential competing interests: Parth Patwari reports a relationship with Takeda Pharmaceuticals USA Inc that includes: employment. If there are other authors, they declare that they have no known competing financial interests or personal relationships that could have appeared to influence the work reported in this paper.

## Data Availability

Data will be made available on request.
