# Supplementary material for: Branched chain amino acids prime metabolic inflammation
Source: Mol Metab. 2025 Dec 15;104:102308. doi: 10.1016/j.molmet.2025.102308 (PMC12818990; doi:10.1016/j.molmet.2025.102308)
Supplement: Multimedia component 1 [file mmc1.pdf]

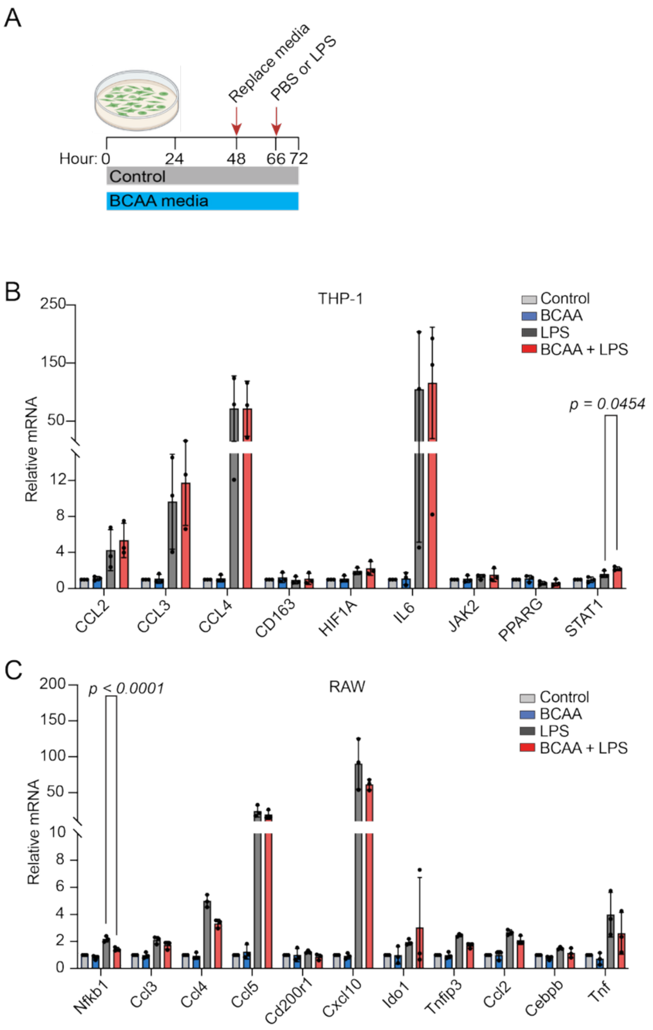

Figure S1: **Inflammatory marker gene expression in THP-1 and RAW 264.7 cells after BCAA exposure**

A. Schematic depicting experimental design. THP-1 or RAW 264.7 cells (n=3 biological replicates per group) were treated with BCAA enriched (5mM) media (BCAA) or control media (Control) for 72 hours with LPS (100 pg/mL) or PBS administered 6 hours prior to cell harvest.
B. Gene expression of inflammatory markers in THP1 cells. Each dot represents a biological replicate. Data normalized to GAPDH and normal media/PBS (Control) values and analyzed with one-way ANOVA with Šidák multiple comparisons test.
C. Gene expression of inflammatory markers in RAW 264.7 cells. Each dot represents a biological replicate. Data normalized to GAPDH and normal media/PBS (Control) values and analyzed with one-way ANOVA with Šidák multiple comparisons test.
D. Gene expression of inflammatory markers in HMC3 cells. Each dot represents a biological replicate. Data normalized to GAPDH and normal media/PBS (Control). Due to skewed distribution, non-parametric ANOVA with Friedman test used.
E. HMC3 cells were cultured in BCAA or BCAA/rapamycin media followed by stimulation for 24 hours with IFNγ (10 ng/mL) like Figure 1D. The most robust signals from Figure 1D were analyzed by qPCR (n=5 biological replicates). Two-way ANOVA was performed to assess the modifying effects of either BCAA or rapamycin with p-values reported in the figures. Rapamycin modulated the expression of all 3 genes but did not attenuate the BCAA effect.

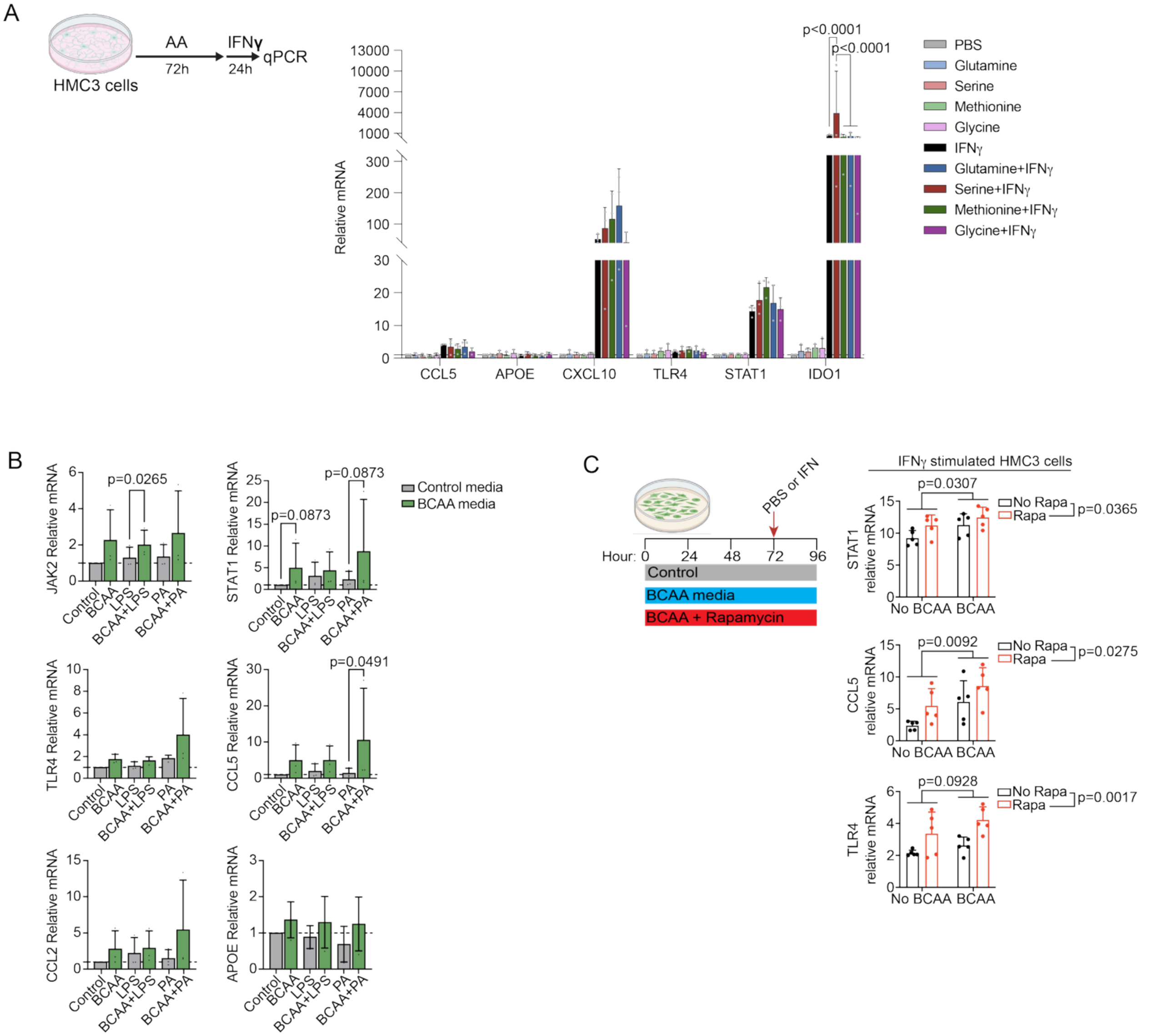

Figure S2: **BCAA modulation of gene expression in HMC3 cells**

A. Following the timeline protocol outlined in Figure 1D, HMC3 cells were cultured in non-BCAA amino acids, followed by IFNγ (10 ng/mL). Unlike BCAA, there were not significant differences in the response to IFNγ, except for the metabolic gene IDO1, the expression of which was potentiated by serine (n=3 biological replicates, ANOVA with Tukey test).
B. Gene expression of inflammatory markers in HMC3 cells. Each dot represents a biological replicate. Data normalized to GAPDH and normal media/PBS (Control). Due to skewed distribution, non-parametric ANOVA with Friedman test used.
C. HMC3 cells were cultured in BCAA or BCAA/rapamycin media followed by stimulation for 24 hours with IFNγ (10 ng/mL) like Figure 1D. The most robust signals from Figure 1D were analyzed by qPCR (n=5 biological replicates). Two-way ANOVA was performed to assess the modifying effects of either BCAA or rapamycin with p-values reported in the figures. Rapamycin modulated the expression of all 3 genes but did not attenuate the BCAA effect.

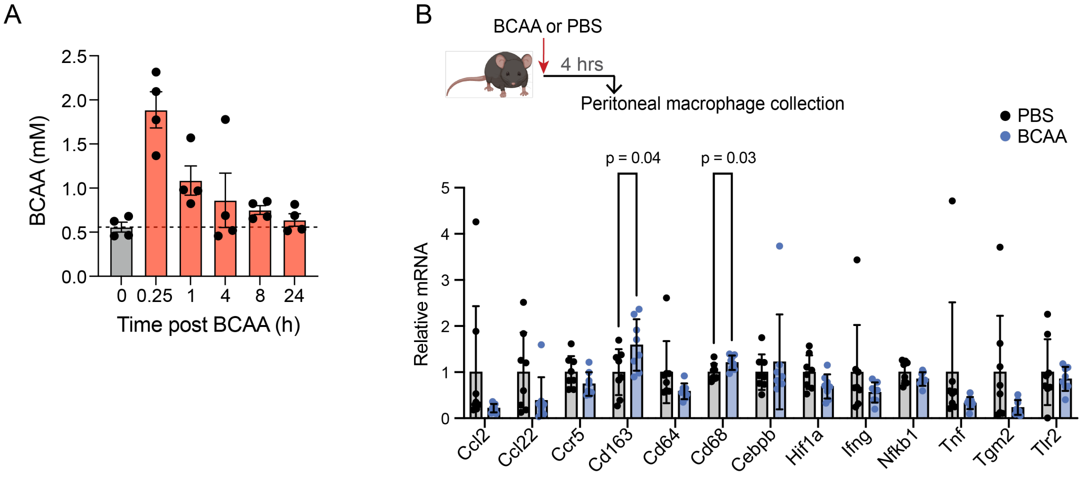

Figure S3: Acute BCAA response in male mice

A. Change in serum BCAA concentration after a single 9 mg dose equimolar BCAA (n=4 mice per timepoint). This dosing approach was used for subsequent *in vivo* murine experiments.

B. Gene expression in peritoneal macrophages harvested 4 hours after single I.P. administration of BCAA or PBS (control). Each dot represents a biological replicate (n=8 male mice/group). Significance assessed for each gene by unpaired t-test.

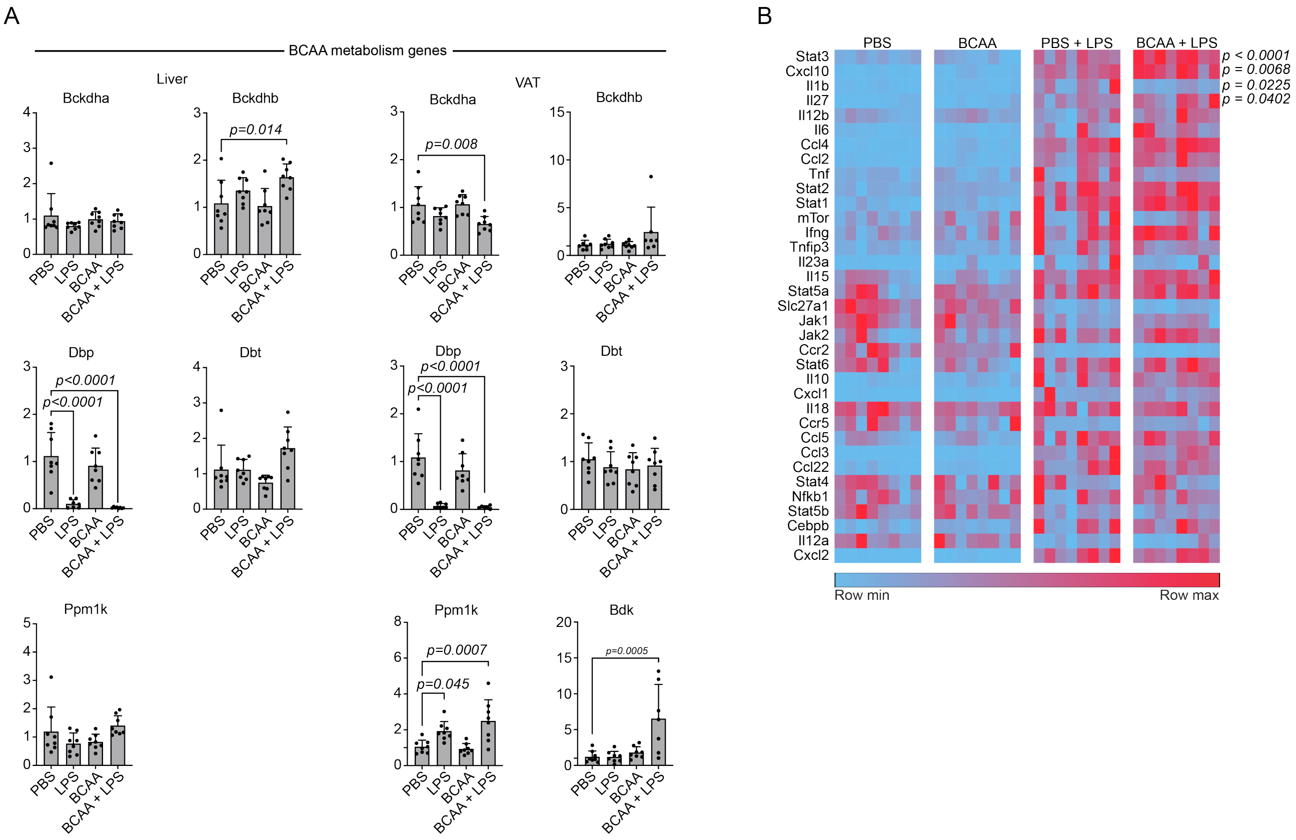

Figure S4: BCAA modulate transcriptional responses to endotoxin.

A. qPCR analysis of BCAA effect relative to PBS (control) on the expression of BCAA metabolic genes in liver (left) and visceral adipose tissue (right). While BCAA had no significant effect alone, BCAA potentiated the expression of a subset of LPS responsive metabolic genes.

B. Heat map showing qPCR analysis of a targeted set of inflammation genes in spleen. Each square represents a biological replicate (n=8 biological replicates per group). Data normalized to *Gapdh* and control (PBS) values and significance assessed with one-way ANOVA with Šidák multiple comparisons test.

A

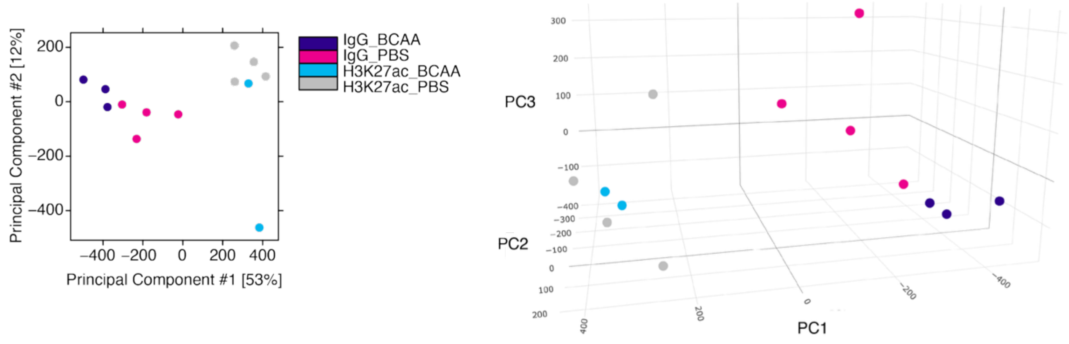

B

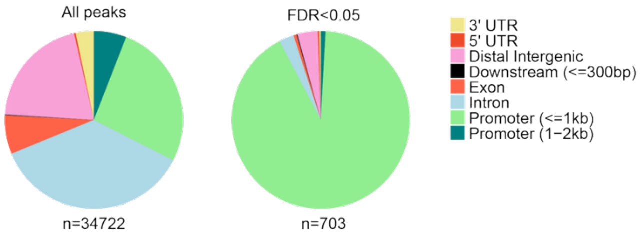

Figure S5: ChIP-seq of murine liver after 5 days administration of BCAA.

A. PCA plot of liver samples that met QC including IgG controls and H3K27ac ChIP for BCAA and PBS (control) treated mouse liver. Right: Greater proximity of BCAA samples (blue) seen with inclusion of PC3.
B. Parts of whole graphs showing total peak distributions (left) with BCAA sensitive peaks that met FDR<0.05 (right).

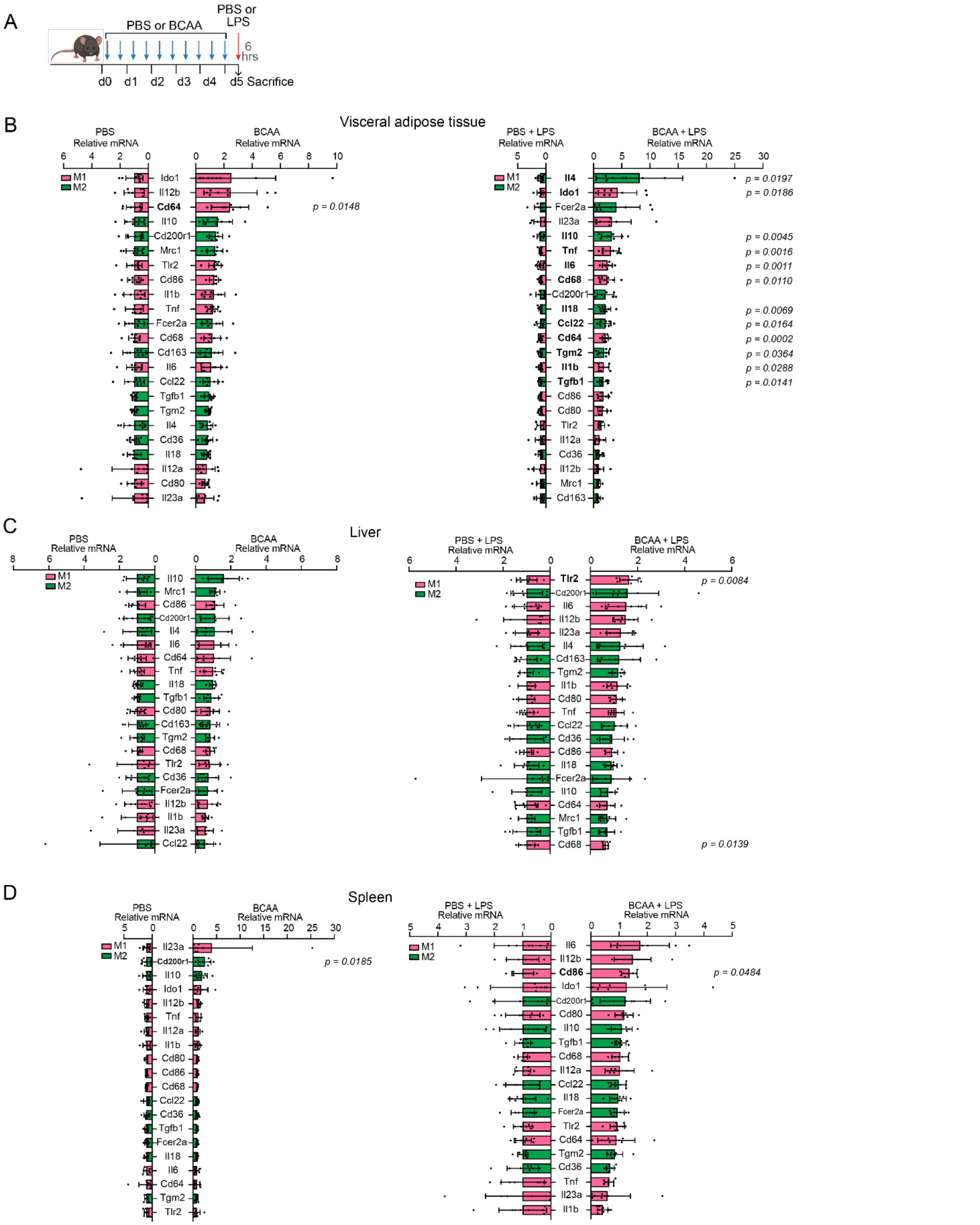

Figure S6: BCAA modulates tissue expression of macrophage markers

A. Experimental schematic related to Figure 2 (n=8 mice per group).
B. qPCR analyses of visceral adipose tissue. Left: BCAA effect alone. Right: BCAA modifying effect on LPS response.

C. qPCR analyses of liver. Left: BCAA effect alone. Right: BCAA modifying effect on LPS response.
D. qPCR analyses of spleen. Left: BCAA effect alone. Right: BCAA modifying effect on LPS response.
